# Blunt trauma to abdominal solid organs: an experience of non-operative management at a rural hospital in Zambia

**DOI:** 10.11604/pamj.2021.38.89.20061

**Published:** 2021-01-27

**Authors:** Sergiy Karachentsev

**Affiliations:** 1 Roan Antelope General Hospital, Luanshya, Zambia

**Keywords:** Blunt abdominal trauma, liver injury, spleen injury, surgery, non-operative treatment, regional hospital

## Abstract

**Introduction:**

although non-operative management of patients with blunt trauma to abdominal solid organs has become standard care, the role of peripheral hospitals remains poorly defined. This study reviews treatment and outcomes in patients with liver and spleen injuries at a regional hospital over a 10-year period.

**Methods:**

a retrospective review of prospectively collected data was performed and supplemented by case notes retrieval. All patients with solid visceral injuries managed between 2009 and 2019 at a rural surgical hospital in Zambia were included. On admission, the patients were offered either urgent laparotomy or non-operative management (NOM) depending on their haemodynamic status. Continuous variables were expressed as median and mean ± standard deviation; categorical data were expressed as percentages. Statistical evaluation of data was performed by two-sample t-test. Statistical significance was assigned at p<0.05.

**Results:**

fourty-three patients were included. The majority of victims sustained isolated spleen or liver injury. Twenty-three patients were urgently operated due to haemodynamic instability. Splenectomy performed in 17 patients, liver laceration sutured in 5 patients. One patient underwent concomitant splenectomy and liver repair. Conservative management was attempted in 20 (47%) patients and was successful in 18 (42%). In two patients NOM failed and splenectomy was performed urgently. Two patients died postoperatively. There were no deaths in NOM group.

**Conclusion:**

NOM of patients with injury to solid abdominal organs could be safely initiated in rural hospitals provided there is uninterrupted monitoring of patients' condition, well-trained staff and unrestricted access to the operating theatre (OT).

## Introduction

Trauma is the leading cause of disability and death in young population both in western world and developing nations [1,2]. Abdominal trauma represents a significant part of these injuries with the spleen and the liver being the most affected organs [3]. Introduction of computerized tomography (CT) scan into management of patients with blunt abdominal trauma (BAT) in the 1980´s improved assessment of intra-abdominal injuries providing a major step towards NOM [4]. At present NOM of spleen and liver trauma is considered to be an initial treatment of choice which obviates non-therapeutic celiotomies and a need for blood transfusion, reduces hospital cost and length of stay [3,5-7].

It is also recommended that conservative treatment of abdominal injuries should be attempted in centres that have experienced surgeons, the capability for precise diagnosing (CT scans), uninterrupted close monitoring in intensive care units and immediate access to the OT [5,7]. However, in a recent study, Tessler RA *et al*. suggested that paediatric patients with isolated low-grade solid organ injury could be stayed for care at a lower level trauma centre [8]. Despite the fact that a good proportion of BAT victims are usually admitted to the peripheral hospitals, the role of these hospitals in the management of abdominal trauma patients still remains unclear. Should rural hospitals be used only as a stage of transportation to dedicated trauma centres or patients happened to be hospitalized to peripheral hospitals could be kept there for the definitive treatment? After all, how safe is it to place patients with BAT on NOM in low-volume surgical hospitals? This study attempts to provide answers to these questions. The objective of the present study was to evaluate the quality of surgical care for patients with solid viscus injury including NOM at a rural hospital.

## Methods

Patients presented with BAT to Roan Antelope General Hospital (RAGH), Zambia in the period from March 2009 to July 2019 were analysed. Those victims sustaining isolated injury to hollow abdominal organs, namely the urinary bladder, uterus and small intestine, were excluded from the study. RAGH is the level II regional hospital having a capacity of 350 beds and operating as the main medical centre in the area with the population of approximately 200,000 people. Confidentiality of all patients´ information was maintained and permission from the hospital ethical committee for the use of medical records data and publishing the study results was obtained.

A retrospective review of prospectively collected data was undertaken and supplemented by retrieval of case notes where necessary. Demographic data, time elapsed from the accident, haemodynamic and physiological parameters, laboratory values, diagnostic examinations, management strategies and outcomes were compiled. Haemodynamic instability was defined as systolic blood pressure (BP) <90mmHg, or >90mmHg but requiring bolus infusions/transfusions and/or vasopressor drugs. Positive abdominal findings included abdominal pain, rigidity of abdominal wall muscles and positive rebound sign on examination. Assessment of extent of the trauma was numerically evaluated according to the injury severity score (ISS) (in patients sustained multiple injuries) [9] and abbreviated injury scale (AIS) using the updated revision of the organ injury scaling committee of the American association for the surgery of trauma [10].

Clinical decision to perform NOM or immediate laparotomy based on assessment of vital signs (BP, pulse and respiratory rate), abdominal examination, urgent laboratory tests (full blood count, blood chemistry) and ultrasound (US) of the abdomen. Patients were assessed by a team comprising of the medical officer on-call, surgeon and anaesthetist. Intravenous (IV) access for fluid therapy was obtained parallel with the examination of the patient within the first 30 minutes of patient entering the emergency department. US was performed by a specially trained staff on admission and thereafter during the hospital stay to determine the organ affected and document the amount of blood in peritoneal cavity. Conventional monitoring included tracking of vital signs, performing serial physical examinations and obtaining several haemoglobin levels. Increased abdominal pain, physical and laboratory findings of acute blood loss, positive abdominal signs and US findings of increased amount of fluid in abdominal cavity indicated on-going (or recurrent) intra-abdominal bleeding and were considered as failed NOM (NOM-F) indicating emergency laparotomy. If immediate laparotomy was opted, a bolus of IV antibiotic (usually 1g of cephalosporine) was given.

Operative management (OM) as a definitive treatment included patients treated with urgent surgery at the arrival in the hospital and those operated for NOM-F. Informed consent was obtained on admission from all patients with essential information given in comprehensible way concerning options for the management and associated risks. Patients were followed up in the surgical clinic during 30 days after the date of discharge. Clinical assessment included laboratory tests and US scan of the abdomen. Measures to prevent malaria infection were discussed. Outcome data included NOM, NOM-F, OM and mortality. Continuous variables were expressed as mean ± standard deviation (SD) and median including ranges; categorical data were expressed as percentages. Statistical evaluation of data was performed by two-sample t-test. A p-value of <0.05 was considered as significant. The 95% confidence interval (CI) was reported when appropriate.

## Results

**Figure 1:**
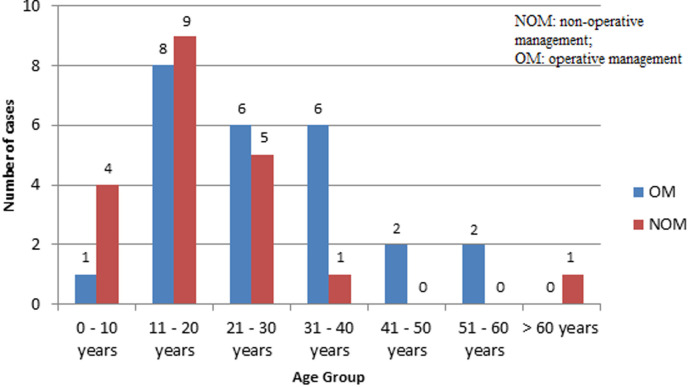
age distribution of patients with blunt trauma to the spleen and liver managed at Roan Antelope General Hospital over a period from 2009 to 2019

A total of 43 patients were included in the study, 34 were men (79%). Age ranged from 6 to 66 years, with a mean age of 23.9 ± 14.4 years. Age distribution is presented in Figure 1. Isolated trauma to abdominal solid organs was noted in 35 patients (81%). In one patient both spleen and liver were lacerated. One patient had associated rupture of the small intestine, another victim sustained concomitant rupture of the gallbladder. Polytrauma was diagnosed in five patients: head injury (N=3), multiple rib fractures and haemothorax (N=1) and fracture of the forearm bones (N=1). ISS for the patients with polytrauma ranged between 17 and 45 with median of 29.0 and mean of 29.4 ± 10.9 with 95% CI 20.0-38.9.

Twenty patients (15 males and 5 females) presenting in stable condition and without signs of peritonism were selected for NOM. Twenty-three patients with clinical signs of haemodynamic instability on admission were directly transferred to the OT for surgical haemostasis. Two patients with failed NOM for rupture of the spleen were also urgently operated. The OM was applied in 58% of patients as a definitive treatment. Demographics of both OM and NOM groups as well as outcomes are listed in Table 1.

All patients entering the OT presented in hypovolaemic shock according to advanced trauma life support (ATLS) classification [11]. Most frequently was recorded class II shock (N=15), followed by class III (N=7) and class IV (N=3). Liver laceration was addressed by suturing of the liver, while patients with spleen rupture underwent splenectomy. In two patients with splenic rupture autotransplantation of spleen tissue was performed into the patch of greater omentum. Interestingly enough, small (about 2.0 and 1.5cm in diameter) additional spleen was found close to the hilum of the organ in two spenectomised patients. It was hypothesized that this tissue could perform the function of the removed spleen to some extent postoperatively reducing the negative effects of splenectomy. As our hospital is not equipped with CT scanner, AIS was calculated assessing depth and extent of organ damage intra-operatively. AIS ranged from 2 to 5 with mean of 3.2 ± 0.8 and 95% CI 2.9-3.5. More than half of the operated patients sustained AIS 3 (14/56%), followed by AIS 4 (5/20%), AIS 2 (4/16%) and AIS 5 (2/8%).

Early post-operative period complicated in two patients by liver hematoma after suturing the liver laceration (N=1) and by respiratory insufficiency suggestive of acute respiratory distress syndrome (N=1) (post-operative morbidity 8%). Both patients were subsequently transferred to the tertiary hospital, the first patient was re-operated 19 days post first operation and the second one received intensive care at intensive care unit (ICU) without re-exploration. Both patients recovered. There were no septic complications of note after surgery. Two patients (8%) died post-operatively: one patient with deeply lacerated enlarged spleen (AIS 4) complicated by massive intra-abdominal bleeding and hypovolaemic shock of class III, died on day 3 post laparotomy; the other victim had laceration to the small intestine associated with liver injury (AIS 3) and hypovolaemic shock III, died on post-operative day 4. There were neither complications nor deaths in NOM group.

## Discussion

This study describes management of blunt liver and spleen injuries including non-operative treatment at a rural surgical hospital. In the last few decades, incorporating NOM into treatment was one of the most notable changes in the care for patients after BAT due to its unequivocal benefits [5,6,12,13]. The advantages of the preserving spleen as an organ of immune system are especially important in the regions with a high incidence of infections such as malaria. In the line of the recent recommendations, an abdominal CT scan is considered to be a gold standard of investigation in haemo-dynamically stable patients with spleen and liver injury [4,6,14]. In reality, peripheral hospitals having no access to interventional radiology often become the first institutions to receive trauma victims and surgeons working in rural areas face the challenges of immediate care for these patients. In particular, surgeons need to solve a dilemma of referring the patients to the trauma centre with the risk of deterioration of their condition during the transfer or keeping them in the ward further taking all responsibility for the outcome. At the same time, search of the literature revealed only two reports about management of blunt trauma to the liver and spleen at peripheral hospitals: the first describes management of paediatric blunt splenic injury at an American adult level II rural trauma centre (2012) [15] and the second is a case report from peripheral Saudi Arabian hospital on the blunt liver trauma (2000) [16]. In this respect the present study addresses the knowledge gap on surgical care in the remote areas and aims to encourage further research in the field. The study itself was stimulated by the experience of having patients with a history of several days trauma in fair general condition, being haemodynamically stable and having documented evidence of injury to solid organs and free fluid (blood) in peritoneal cavity by ultrasound (US) scan. According to the current therapeutic approach, these patients were offered NOM with continuous monitoring of vital signs, laboratory data and repeated US scan. Patients with unstable cardio-vascular status were urgently operated with liver laceration having sutured or spleen removed.

In this study, the cohorts of NOM and OM were different in terms of age (p<0.05) and time since injury (p<0.02) (Table 1). Patients in the NOM group appeared to be younger than those in OM cohort and this reflects the well-known fact of children having more chances of successful NOM [5,8,15,17]. Late presentation was recorded for both groups, but it was especially common in the NOM group. This could be explained by the fact that our hospital receives most of the patients from two district hospitals and the local clinics in the area. In cases of ongoing intraabdominal bleeding significant delays in admission could worsen the patients´ condition and pose a serious challenge to the surgeon. On the other hand, when the patient appears to be haemodynamically stable one or two days after the injury, the choice of NOM becomes more evident and safe.

**Table 1:** patient demographics and outcomes

Characteristics	OM (N=25)	NOM (N=18)	p value
Age (years), mean ± SD	27.9 ± 14.0	18.2 ± 13.4	<0.05
Median (range)	23.0 (6-59)	14.5 (8-66)	
M/F	21/4	13/5	
Length of history (hours), mean ± SD	16.2 ± 16.7	33.1 ± 27.9	<0.02
Median (range)	12.0 (2-72)	24.5 (4-96)	
Spleen injury	19	15	
Liver injury	5	3	
Spleen + liver injury	1	0	
AIS	3.2 ± 0.8	undefined	
Morbidity	2/8%	0%	
Mortality	2/8%	0%	

OM: operative management; NOM: non-operative management; SD: standard deviation; M/F: males/females; AIS: abbreviated injury scale

It is not uncommon that BAT is associated with other injuries such as orthopaedic trauma, head injury or vascular trauma [6,12,13,17] which add morbidity and mortality in trauma patients [6,12,18]. By contrast, the presented study revealed as many as 88% of the patients sustained isolated trauma to abdominal organs. These findings could be attributed to a high proportion of children in this study (20 patients, 46.5% were below 18 years old) sustaining a low-energy trauma by falling from the low height, usually a tree. Low incidence of polytrauma in our study was considered as a favourable factor, because: abdominal trauma is associated with lower morbidity and mortality if no other organ seriously injured [18]; the risk of missing an associated abdominal hollow viscus organ injury in monotrauma patients is less [18,19]. As a result, NOM in single abdominal injury is more effective [19].

The proportion of NOM in the abdominal trauma patients reported in the literature varies significantly from 40% to as high as 95% [3,6,12,17,19,20]. While haemodynamic status of the patient on arrival to the hospital is considered to be the most important factor for employment of NOM pathway [3,6,7,20], there are other elements influencing the treatment choice such as the anatomy of the injury [5,12], age of the patient [5,13] and the presence of associated intra-abdominal and extra-abdominal trauma [7,12,13,19]. One should also consider the organizing and structural abilities of the hospital to access the OT in the shortest time and to utilize the most modern diagnostic instruments to offer non-operative options such as the splenic artery embolization [7,12,13]. In this study, 42% of the patients with blunt trauma to abdominal solid organs were treated conservatively. It reflects the real situation with abdominal trauma in Zambia and gives a clear room for improvement. At the same time, high success rate of initiated NOM (18 out of 20 patients, 90%) justifies the policy adopted in our institution.

Therefore, certain factors like predominance of isolated abdominal trauma, significant percentage of paediatric injuries and high proportion of late presentations make it possible to initiate and safely provide NOM for patients with blunt spleen and liver injuries at a regional low-volume surgical hospital which orientates towards the current principles of management of surgical patients.

**Limitations:** this observational retrospective study was performed in a low-volume hospital with small size of patients´ cohorts that appeared to be not ideally statistically comparable. Search of the literature and all the procedures were performed by the sole researcher which can lead to errors in the search protocol and intervention bias. Due to the lack of CT scanner it was not possible to calculate AIS in the cohort of non-operated patients. As a result, the author could not compare the extent and depth of organ injury in presented two groups of patients. All these limitations can reduce internal and external validity of the research. However, the author believes that a systematic approach to the literature search and rigorous data extraction employed as well as evidence-based and time-proven operative technique used have not compromised the results of the study.

## Conclusion

Blunt abdominal trauma remains a common phenomenon and a serious problem for healthcare institutions worldwide and hospitals operating in rural areas are not exception to this rule. This single institution study analyzed cases of blunt trauma to abdominal solid organs among the rural population in Zambia. NOM could be initiated in a low-volume regional surgical hospital provided the proper selection of the patients is performed, constant monitoring of the patients´ condition is ensured, appropriately trained staff is available and the OT is ready round the clock for urgent laparotomy. Larger-scaled and prospective studies are needed to validate the findings obtained in this study and develop practical recommendations for surgeons working in rural areas.

### What is known about this topic

- The management of abdominal solid organs trauma has changed considerably in the last few decades in favour of NOM;
- NOM of blunt hepatic and splenic injuries is currently the treatment modality of choice in haemodynamically stable patients;
- Decision between NOM and OM depends on careful risk-benefit analysis for each patient and should be a product of the multidisciplinary team work.

### What this study adds

- NOM could be safely initiated at regional rural hospitals under the conditions of constant monitoring of vital signs, high level of expertise of the surgical team and unlimited access to the OT;
- Late admissions of the patients with BAT should be addressed promptly by rising awareness of the problem in the community and educating our colleagues in district hospitals and local clinics to identify the abdominal injuries early and refer the victims without delay.
